# A Few Thoughts on Filling Teeth

**Published:** 1879-10

**Authors:** T. H. Parramore


### ARTICLE II.

### "*A Few Thoughts on Filling Teeth.*"

BY T. H. PARRAMORE, D. D. S.

The first and most important question that presents itself to the mind of every honest practitioner of Dentistry is, how he can best and most faithfully meet the demands of those who have entrusted themselves to his care, and save for the longest time those organs so essential to health and happiness, the teeth.

Until in coming ages science shall have furnished us a material or process by which those substances so destitute of live material, (enamel and dentine,) shall be enabled successfully to combat the encroachments of decay, and repair the injury already done; the dentist's principle dependence is in *filling.* Therefore, this question of *filling* is to the dentist the most important with which he has to deal. Filling teeth is necessarily, in a great measure, a mechanical process, but in order to be a successful *filler* of *teeth,* a man must be thoroughly acquainted with physiological and pathological conditions, as these are the indices that point out the material suitable for each case. In my opinion no dentist can afford to dispense with any one of the standard materials at present presented to the profession for the purpose of filling.

The first thing to be considered is the character of the tooth or teeth to be operated upon, age, sex, and physical condition of patient, and (which is often a very important question,) can we see and have control of the teeth in the future as often as we deem necessary. If the teeth belong to what is known as class first, ("The Principles and Practice of Dental Surgery"—Part II., Ch. II.,) and there are no well-defined physiological or pathological conditions present, rendering such practice unsafe, gold is the best material indicated. In patients with teeth of this class, symptoms that would render filling with gold unsafe are rarely found, but sometimes in the case of females during the period of pregnancy and lactation, or in the case of young patients (principally females,) the teeth are found so sensitive as to render the use of so good a conductor of heat and cold unsafe. The thermal changes produced by putting so fine a conductor in contact with irritated dentine, may result in inflamation of a serious character.

In the first of these cases the trouble ends when the period of lactation is completed, the second proceeds from troubles often in organs remote from the teeth, and depend upon their restoration to health for its removal. The best practice in such cases is to fill with oxy-chloride of zinc or gutta percha, until hypersensativeness is relieved, then fill permanently with gold. These rules apply to the other classes described in the chapter above referred to; they, however, require greater care and a longer time, and in some cases it is never safe to fill with gold or amalgam. In some other cases (this rule is specially applicable to young patients,) after being filled for years with gutta percha, teeth at first unfit for such a filling will be so greatly improved by the deposition of mineral matter as to have a metalic filling. In very many cases in all classes of teeth, gold is the very best material to use; but in teeth of an unhealthy blueish or white chalky character with very thin friable walls, or in mouths where a decided acid reaction exists, gold should not be used.

Gold is a metal upon which the oral secretions have absolutely no effect; it does not corrode, does not shrink, and, therefore, when well inserted makes a perfectly water-tight filling. It is sufficiently hard to bear the mar of mastication; its color is but slightly objectionable, it works easily when properly treated, and when used discreetly is decidedly the *very best* material dentists have at their command. It has its objectionable points, however, first among which I consider its conductile properties, both thermal and electrical. The first would render it an unsafe material to use upon hypersensitive dentine or near an exposed nerve. When two materials of unequal electric conductility are in contact, the electric fluid acts upon and disintegrades the poorer conductor. As stated above, *gold* is a very fine conductor of electricity, and it is well-known that tooth substance is a very poor conductor of electricity, therefore, when teeth (in mouths where such a condition exists or is apt to exist), are filled with gold, we are apt to see them decaying around the gold and the fillings soon dropping out. In what mouths are electrical influences apt to exist? In deciding this question we must be guided by the appearance of the teeth, gums, and mucous-membrane of the mouth and chemical condition of the oral secretions.

Next in order to gold as a filling material, I would place amalgam. The profession is too well acquainted with the material sold as amalgam for it to require any description or definition at my hands. It should be used with care and discretion, and when so used, fills a place at present occupied by no other material known to the profession. It works easily, sets quickly, making a filling sufficiently hard to withstand the greatest amount of mastication; can be readily packed against the most frail walls, (which is often a very difficult and hazardous undertaking with gold;) it is a material with which we can often save for a long time the teeth of poor patients unable to pay for expensive gold fillings, it serves a good purpose in building crowns upon old molar roots, a work that would require hours with gold but can be accomplished in a few minutes with amalgam, and often lasts longer than such a work of gold. There are places in the mouth when the best operators often fail to put in perfect gold fillings; in such a place an average dentist would put in a first-class amalgam filling, which would be decidedly better than an imperfect gold filling. It often answers a good purpose in permanent teeth of children, to remain until they grow old enough to bear the more tiresome operation of filling with gold. There are, however, many serious objections to this in many respects admirable material. Its color is at best bad, turning in some mouths perfectly black ; it has a tendency to draw away from the walls of a cavity or what is called "ball ;" this tendency can be generally obviated by observing proper care in its insertion. The objection urged against gold on account of its conductile properties applies to amalgam, but in a modified degree.

There is no material known to the dentist, that in so many respects measures up to his ideal filling material as gutta percha. Gutta percha is a vegetable production, but is prepared for the dentist's use by mixing with it carbonate of lime, quartz, etc. It is a poor conductor of heat and about as good a conductor of electricity as tooth substance; it is non-irritant; has a good color, makes a water-tight filling when properly inserted, and has but one serious defect that I know of, and until this is overcome it can never be considered more than a temporary filling material. It is so soft that it wears away rapidly. But with this defect it has in many cases, such as in children's teeth and the teeth of young persons where there is a great deficiency of mineral in proportion to animal matter in cases of hypersensitiveness of dentine, as a non-conducting substance under gold fillings in cases of a nearly exposed nerve, etc., no equal. I use no other material for filling the front teeth of many of my patients whom I can have come to my office as often as I desire, adding more gutta percha as fast as the filling wears away.

There are a great many preparations of oxy-chloride of zinc, known by different names, but they are essentially the same. It should only be used (in which case it has no equal) as a capping for exposed nerves, together, but not combined with lacto phos. lime, or by mixing the oxide with creasote or oil of cloves. Place in contact with exposed nerve and fill cavity with oxychloride, after which remove part of the oxy-chloride filling and fill permanently. The principle reasons for not using oxychloride as a permanent filling are, it is subject, on account of its softness to the objection urged against gutta percha, and it softens, and disintegrades in all mouths after a while, and in most mouths very quickly.

If I have succeeded in demonstrating that each case claiming the attention of the dentist requires special treatment, and shall arouse to a closer study of this subject so important to our progress as a profession, one earnest worker, I shall feel richly compensated for the labor it has cost me.

